# Maximising response to postal questionnaires – A systematic review of randomised trials in health research

**DOI:** 10.1186/1471-2288-6-5

**Published:** 2006-02-23

**Authors:** Rachel A Nakash, Jane L Hutton, Ellen C Jørstad-Stein, Simon Gates, Sarah E Lamb

**Affiliations:** 1 Warwick Emergency Care and Rehabilitation, Warwick Medical School, Gibbet Hill Campus, University of Warwick, CV4 7AL, UK; 2 The Kadoorie Centre, John Radcliffe Hospital, Headley Way, Headington, Oxford, OX3 9DU, UK; 3 Department of Statistics, University of Warwick, CV4 7AL, UK

## Abstract

**Background:**

Postal self-completion questionnaires offer one of the least expensive modes of collecting patient based outcomes in health care research. The purpose of this review is to assess the efficacy of methods of increasing response to postal questionnaires in health care studies on patient populations.

**Methods:**

The following databases were searched: Medline, Embase, CENTRAL, CDSR, PsycINFO, NRR and ZETOC. Reference lists of relevant reviews and relevant journals were hand searched. Inclusion criteria were randomised trials of strategies to improve questionnaire response in health care research on patient populations. Response rate was defined as the percentage of questionnaires returned after all follow-up efforts. Study quality was assessed by two independent reviewers. The Mantel-Haenszel method was used to calculate the pooled odds ratios.

**Results:**

Thirteen studies reporting fifteen trials were included. Implementation of reminder letters and telephone contact had the most significant effect on response rates (odds ratio 3.7, 95% confidence interval 2.30 to 5.97 p = <0.00001). Shorter questionnaires also improved response rates to a lesser degree (odds ratio 1.4, 95% confidence interval 1.19 to 1.54). No evidence was found that incentives, re-ordering of questions or including an information brochure with the questionnaire confer any additional advantage.

**Conclusion:**

Implementing repeat mailing strategies and/or telephone reminders may improve response to postal questionnaires in health care research. Making the questionnaire shorter may also improve response rates. There is a lack of evidence to suggest that incentives are useful. In the context of health care research all strategies to improve response to postal questionnaires require further evaluation.

## Background

Numerous market and educational research studies have been carried out to evaluate strategies of improving response rates to postal questionnaires. However, none have been specific to the health care setting, nor to the context in which participants are receiving or being allocated an experimental health care treatment [1-5]. A Cochrane review has been undertaken and recently updated but is not restricted to health care studies [1]. The majority of the trials in the Cochrane review (approximately 80%) were published in market research or educational research journals and had no health care connection. The generalisability of the results of this review into the health care setting has been questioned [6]. The need for reviews focussing on patient populations and health care studies is well recognised [7,8]. Good quality clinical trials often recruit many thousands of patients to detect clinically relevant treatment effects [9]. Patient surveys are also a valuable way of obtaining data in health care research. Postal self-completion questionnaires offer one of the least expensive modes of collecting patient based outcomes in large target groups [10]. A major disadvantage with postal questionnaires, however, is non-response (or loss to follow-up). This reduces the effective sample size and may introduce bias [11,12]. Identifying and implementing effective methods to promote follow-up is an essential component of study design and management. We conducted a systematic review to identify effective methods of improving response to postal questionnaires in patient populations recruited to health care research activities.

## Methods

A systematic review with a meta-analysis.

### Search strategy

Randomised trials of methods of improving response to postal questionnaires in health care research were identified. Seven electronic bibliographic health care and medical databases were searched for relevant trials (Table 1). The reference lists of identified trials and reviews were also searched. Authors of relevant trials and reviews were contacted to identify unpublished trials. Selected journals were hand searched. The BMJ 'Cite Track Alert' service [13] was used to alert for articles citing the most recent relevant review [1] and the 'Biomail' Medline search service [14] was used with the search terms of ('clinical trial') and ('follow-up' or 'questionnair*'). There were no language restrictions.

**Table 1:** Electronic bibliographic databases searched and search strategy used

*Database*	*Host*	*Search Strategy*
Medline (1996–2004)	Ovid	**1. **Health care survey* *or *Questionn* 2. Respons* *or *Respons* adj rate *or *follow adj up *or *return **3. **Post* *or *mail* 4. Enhanc* *or *improv* *or *promot* *or *increas* *or *influenc* *or *maximis* 5. Remind* *or *letter* *or *postcard* *or *incentiv* *or *reward *or *money *or *payment *or *lottery *or *prize *or *personalis* *or *sponsor *or *length *or *style *or *format *or *appearance *or *colour *or *color *or *stationary *or *envelope *or *stamp *or *postage *or *certified *or *registered *or *telephone *or *notice *or *dispatch *or *deliver *or *sensitive *or *disseminate **6. **Randomi* *or *control* *or *trial* 7. 1 *and *2 *and *3 *and *4 *and *5 *and *6
Embase (1980–2004)	Ovid	
CENTRAL (1980–2004)	Update Software ltd	
Cochrane database of systematic reviews (1980–2004)	Update Software ltd	
PsycINFO (1990–2004)	Ovid	
National Research Register (2000–2004)	DoH (Web version)	

### Study selection

All identified randomised trials of any method of improving response to postal questionnaires in a health care context were evaluated for study inclusion. 'Health care research' is defined as the questionnaire being used in a clinical trial, survey or observational study of health state and containing questions relating to aspects of a person's physical, mental or social well-being (based on the WHO definition of health[15]). Only studies that recruited patient populations were included. A 'patient' is defined as a person who is receiving medical or surgical treatment [16]. Studies in which participants were recruited via GP patient lists but were not actively receiving medical treatment were excluded. A list of excluded studies is available from the authors. The criterion to assess the effect of the interventions was a comparison of the percentage of questionnaires returned after all follow-up efforts. All potentially relevant studies were checked for study quality independently by two reviewers.

### Quality assessment

Quality assessment was based on recommendations in the Cochrane Reviewers Handbook [17] and a Delphi List of quality criteria developed by Verhagen et al[18]. Where aspects of quality were unclear from the report the authors were contacted for clarification.

### Data extraction

Data were extracted independently by two reviewers using a standard data extraction form. Details extracted included the country, main study method, patient characteristics, intervention used to improve response, number of participants randomised to the intervention and control groups and response rate in terms of number and percentage of questionnaires returned and procedures for follow-up. Where insufficient data were reported the authors were contacted for clarification. When studies used more than two categories to evaluate an intervention, (for example short, medium and long questionnaires) a dichotomy was created by combining the categories that were most similar. When this has been done it is indicated on the Data Extraction table (Additional file 1).

### Quantitative data synthesis

The results were pooled into sub-groups of similar interventions. The data were analysed using the Cochrane review manager software (RevMan version 4.2; Oxford, UK). We used the Mantel-Haenszel method to calculate the pooled odds ratios (OR) for binary outcomes for each strategy. This fixed effect method based on a weighted average of the results was used to combine studies. A sensitivity analysis was carried out by re-analysing the data using a random effects model. For all estimates we calculated 95% confidence intervals (CI 95%). Statistical heterogeneity between trials was assessed with χ^2 ^tests using P < 0.10 to reflect significant heterogeneity and the percentage of variation across the studies was measured using the I^2 ^statistic [19]. Publication bias was investigated using a funnel plot.

## Results

### Trial flow

**Figure 1:**
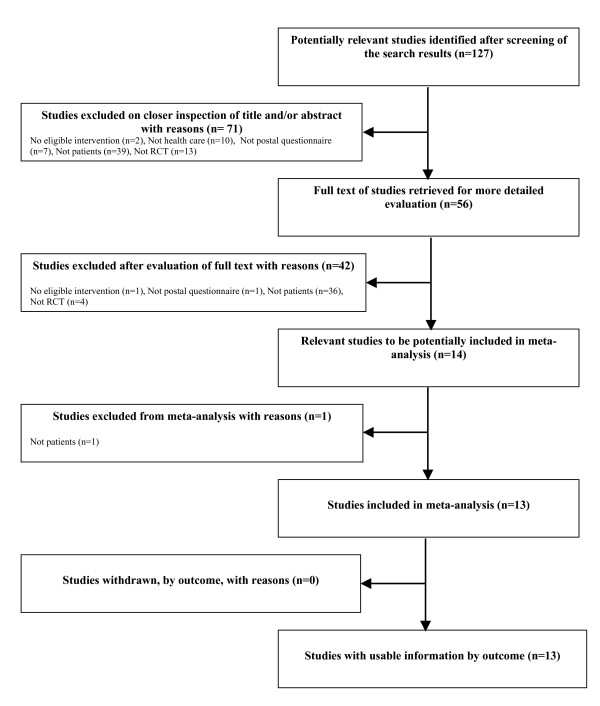
Flow diagram of study selection process.

We identified 13 randomised trials including 25607 participants that fulfilled our inclusion criteria [20-32]. Figure 1 gives a flow chart summarising the study selection process.

### Study characteristics

The studies evaluated five different methods of enhancing response to postal questionnaires. These methods were: questionnaire length, incentives (cash, prize draw, lottery or phone card), question order, reminder strategies and including an information brochure with the questionnaire. One paper reported results in two distinct patient groups (angina and asthma) and these are presented as separate studies [28]. Another paper described two separate interventions (questionnaire length and incentives) and these are also reported as separate studies [24]. Six papers contained information regarding missing data from the returned questionnaires [20-23,28] but used different interpretations of missing data. All the studies incorporated their randomised trial of methods of improving response into an existing research study. The majority of the studies nested their trial of enhancing response within a patient survey. None of the studies nested their study of methods of improving response into a randomised clinical trial. Additional file 1 gives details of extracted data. Five studies were deemed to be of 'good' quality, six were 'moderate' quality and quality was unclear from the report of four studies. See Table 2 for details of quality assessment.

**Table 2:** Quality assessment scores of included studies

Author	Random-isation performed?	Allocation concealed?	Similar baseline characteristics?	Eligibility criteria specified?	Blind outcome assessment?	Adequate reporting of results?	ITT analysis?	No performance bias?	Quality score
Dorman 1997	√	?	√	√	?	√	?	√	A
Dunn 2003	√	?	√	√	√	X	√	√	A
Evans 2004	√	?	√	√	?	X	?	√	B
Iglesias 2000	√	√	√	X	√	√	√	√	A
Jenkinson 2003	√	?	?	√	?	X	?	√	D
Jones a,b 2000	√	?	?	X	?	X	?	√	D
Leigh Brown 1997	√	?	?	√	?	√	?	√	B
McColl a,b 2003	√	?	√	√	X	√	√	√	A
Parkes 2000	√	?	?	√	?	√	?	√	B
Salim Silva 2002	√	?	?	X	?	√	√	√	B
Sutherland 1996	√	?	√	√	?	X	?	√	B
Tai 1997	√	?	√	X	?	√	?	√	B
Ward 1996	√	?	?	X	?	X	?	√	D

To score:

5 or more √ = Good: A

2–4 √ = Moderate: B

4 or more X = Poor: C

4 or more ? = Unclear: D

### Quantitative data synthesis

**Figure 2:**
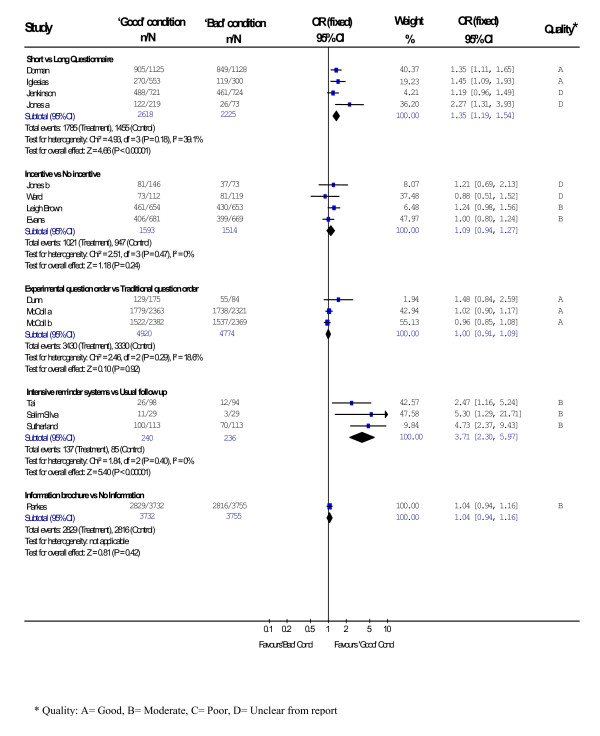
Meta-analysis of methods of improving response rates to postal questionnaires in health care research.

Figure 2 shows the pooled odds ratios and 95% confidence intervals for the five different strategies investigated for improving response rates. Reminder systems had the most significant effect on response rates (OR 3.71, CI 95% 2.30 to 5.97 p = <0.00001) with more intense methods improving response by an average of 24%. Shorter questionnaires improved response rates but to a lesser degree (OR 1.35, CI 95% 1.19 to 1.54 p = <0.00001) with an average improvement in response of 9%. 'Shorter' questionnaires ranged from seven to 47 questions and 'longer' questionnaires ranged from 36 to 123 questions. The studies investigating questionnaire length compared two or more questionnaires. We used the authors own categorisation of 'shorter' and 'longer' questionnaires. The use of incentives (OR 1.09, CI 95% 0.94 to 1.27 p = 0.24), re-ordering of questions (OR 1.00, CI 95% 0.91 to 1.09 p = 0.92) and including an information brochure with the questionnaire (OR 1.04, CI 95% 0.94 to 1.16 p = 0.42) had no significant effect on response rates.

There was no evidence of significant heterogeneity between the trials in each intervention group. Sensitivity analysis using a random effects model gave virtually identical overall estimates of effect.

## Discussion

The main findings are that the implementation of more intense follow-up strategies and shorter questionnaires can improve response rates. In comparison to meta-analyses in non-patient populations our findings show a greater effect size [2-5]. The results are more relevant to health care researchers than previous reviews. Since the most recent previous review [1] we included five new relevant studies.

As with all systematic reviews there is the potential for bias. Studies reporting positive effects are more likely to be published and therefore selected for inclusion in the review. We found evidence of publication bias as the funnel plot was asymmetrical. Re-running all the analyses excluding the smaller studies, however, had little effect on the overall results.

### Population and context for the review

Losses to follow-up in health care research can have serious effects on study validity [33]. A recent Cochrane review [1] identified 292 eligible randomised trials of methods of improving response rates to postal questionnaires. The review concludes that methods such as unconditional incentives (ie incentives given regardless of whether the questionnaire is actually returned), shorter questionnaires and "user-friendly" questionnaires can substantially improve response rates. Caution should be taken when interpreting the findings of this review in a health care context as the majority of the included trials had no health care connection. The motivation of a patient to respond to a follow-up questionnaire in a health care study might differ from that of a member of the public selected to receive a general survey questionnaire. Tactics to encourage response may therefore differ. Health care study participants are actively involved in the research process and are often motivated by the potential health benefits associated with the study. Conversely, the amount of trauma and discomfort produced by the study treatment or procedures affects the willingness of the patient to remain under follow-up [34]. Ludemann et al. [35] found that patients in a clinical trial of laparoscopic fundoplication were less likely to respond to postal follow up if they had a poor outcome from the surgery. Saliency of a questionnaire to the recipient has been shown to be one of the strongest predictors of response. A salient topic is defined as "one which deals with important behaviour or interests that are also current" [36]. It is likely that a participant in a health care study receiving a questionnaire regarding their response to a treatment intervention, or their views on a therapeutic encounter, would find the questions highly salient. Response rates to non-salient questionnaire surveys of the general population rarely exceed 50% [37]. The average response rate across the included studies in our review (excluding two studies that only randomised non-responders to previous follow-up methods) was 65%.

### Follow-up strategies

Three studies investigated methods of follow-up to improve response [29-31]. Although the methods of follow-up differed, all of the trials compared a more intensive follow-up procedure with a standard method. The three included trials compared telephone, postal or recorded delivery reminders compared to usual follow-up efforts. We therefore carried out an analysis of intensive versus usual follow-up. The results suggest that increased intensity of follow-up effort may improve response rates, but the differences between the interventions of the studies in this analysis mean that the result should be treated with caution. In one study [29] the use of telephone reminders appeared to be less effective than recorded delivery postal reminders. However, in another study [30] telephone reminders appeared to be more effective compared to normal delivery postal reminders alone. One of the studies had a very small sample size [30] but excluding this study and re-running the analysis had little effect on the results. Clinical researchers need to incorporate appropriate follow-up strategies within the budget constraints of their research activities. Due consideration for the patients' privacy is needed, however, to ensure that patients do not feel harassed by the follow-up efforts. Further research is required to determine the acceptability of repeated contact to the patient.

### Questionnaire length

A recent review focuses on the effect of questionnaire length on response [38]. Out of twenty seven included trials, fourteen (52%) studied health related topics but only four (15%) studied patients rather than members of the general public. The authors extrapolate that shorter questionnaires should be used in clinical trials to improve response. Since none of the included studies looked specifically at clinical trials, such extrapolation should be viewed with caution. Our findings confirm that shorter questionnaires improve response in the health care setting. Questionnaires are often used in health care research to answer a research question. There is, however, an inevitable trade off between making the questionnaire comprehensive enough to answer the question adequately, and making it so long that it has an adverse effect on response. Careful consideration of the minimum data required when designing the questionnaire is essential. As yet there is insufficient evidence to suggest an optimal questionnaire length in terms of number of questions or pages.

### Incentives

Previous reviews looking predominantly at market research found incentives to be a useful way of improving response [1-3]. The largest effect sizes are seen with monetary incentives. The use of incentives in health care research in Europe is uncommon. Trials often have strict budget constraints making the provision of incentives an unacceptable additional cost. Providing incentives in health care research can also raise ethical concerns [39]. The health care study participant may view their personal input into the study as the motivator to respond rather than merely responding to an incentive. This review has shown no evidence that incentives are effective in the health care context. This is an area, however, which requires further investigation. The studies included in this review used either small monetary incentives or monetary equivalent incentives (lottery ticket, prize draw or phone card). None of the studies investigated non-financial incentives such as pens. The inclusion of an incentive appropriate for the particular study may have a positive effect on response but this has not been tested. Until this area is investigated more fully no recommendations can be made on including incentives in health care research as a method of improving response.

### Question order

Question order appeared to have little effect on response rate. The three studies looking at question order, however, investigated two different approaches. One study compared a traditionally ordered questionnaire with a chronologically ordered one [22] and the other two studies compared placing condition specific questions either before or after generic questions [28].

### Future research

This review was strict in its definition of a 'patient' and excluded studies which were in the health care setting but involved the general public. It was anticipated that more studies would be found involving patients. The evidence available on which to base conclusions was therefore limited. The review could be repeated including health care research studies of the general public to give a broader perspective of methods of improving response in the health care setting. Previous studies have investigated this area evaluating methods of improving response such as postage stamps [40] and questionnaire length and incentives[40,41]. The market research literature has investigated many methods of improving questionnaire response. Edwards et al. [1] grouped these methods into the following strategies: Incentives, Questionnaire length, Appearance, Delivery, Contact, Content, Origin and Communication. All these methods need to be tested on patients in the health care setting before extrapolations of their usefulness can be made. All of the trials included in our review looked at the effect of an intervention in isolation of other interventions. Future studies could use factorial designs to investigate the addition of different methods to improve response.

In any future research it is important that the methods of improving response are well documented and tested in situations that reflect their intended use ie patient populations in health care studies. The effects of the interventions on completeness of the returned questionnaires also requires investigation.

## Conclusion

There is limited evidence of methods to improve response to postal questionnaires in patient populations in health care research. Caution should be taken in utilising the results of previous reviews in clinical study design. Follow-up strategies in the form of repeat mailing or telephone contact offer the most promising method of maximising response to postal questionnaires in health care research. The acceptability of repeated patient contact and ethics relating to this, however, need to be investigated further and guided by research ethics committees. Reducing the length of the questionnaire may also have a positive effect on response.

## Competing interests

The author(s) declare that they have no competing interests.

## Authors' contributions

No persons apart from the authors contributed to this paper. The guarantors of this paper are RN and SL. RN, SL and JH had the original idea for the paper, RN performed the literature search and wrote the paper, RN and EJ conducted quality assessment and data extraction. The paper was drafted by RN and critically appraised for intellectual content by SL, JH, SG and EJ. RN, JH and SL were involved in interpretation of the data. The final version of the paper was approved by all authors.

## Pre-publication history

The pre-publication history for this paper can be accessed here:



## Supplementary Material

Additional File 1 Extracted data of randomised trials of methods of improving response rates to postal questionnaires in health care research. Extracted data from included studies of systematic review. Click here for file

## References

[B1] Edwards P, Roberts I, Clarke M, DiGuiseppi C, Pratap S, Wentz R, Kwan I (2002). Increasing response rates to postal questionnaires: systematic review. BMJ.

[B2] Yammarino FJ, Skinner SJ, Childers TL (1991). Understanding Mail Survey Response Behaviour - A Meta-Analysis. Public Opinion Quarterly.

[B3] Fox RJ, Crask MR, Kim J (1988). Mail Survey Response Rate: A Meta-Analysis of Selected Techniques for Inducing Response. Public Opinion Quarterly.

[B4] Harvey L (1987). Factors Affecting Response Rates to Mailed Questionnaires: A Comprehensive Literature review. Journal of the Market Research Society.

[B5] Kanuk L (1975). Mail Surveys and Response Rates: A Literature Review. Journal of Marketing Research.

[B6] Smeeth L, Fletcher AE (2002). Improving the response rates to questionnaires. BMJ.

[B7] McColl E, Jacoby A, Thomas L, Soutter J, Bamford C, Steen N, Thomas R, Harvey E, Garratt A, Bond J (2001). Design and use of questionnaires: a review of best practice applicable to surveys of health service staff and patients. Health Technol Assess.

[B8] O'Cathain A (2002). Further Analyses Would Make the Review More Helpful - Rapid Response to Edwards et al Increasing Response Rates to Postal Questionnaires: Systematic Review. BMJ.

[B9] Yusuf S, Collins R, Peto R (1984). Why do we need some large, simple randomised trials?. Stat Med.

[B10] Maxim PS (1999). Quantitative Research Methods in the Social Sciences.

[B11] Schulz KF, Grimes DA (2002). Sample size slippages in randomised trials: exclusions and the lost and wayward. Lancet.

[B12] Armstrong BK, White E, Saracci R (1995). Principles of exposure measurement in epidemiology. Monographs in Epidemiology and Biostatistics.

[B13] BMJ Cite Track Alert. http://bmj.com/cgi/alerts/ctmain.

[B14] BioMail Medline Search.

[B15] McCaul LA, Cooper PG (1979). Techniques to increase the response rate in follow-up studies: results of a pilot test. Int J Rehabil Res.

[B16] Anderson S, Carey L, Cullen K, Flackett S, Grandison A (1998). The Chambers Dictionary.

[B17] Clarke M, Oxman AD (2003). Assessment of Study Quality. The Cochrane Library.

[B18] Verhagen AP, de Vet HC, de Bie RA, Kessels AG, Boers M, Bouter LM, Knipschild PG (1998). The Delphi list: a criteria list for quality assessment of randomised clinical trials for conducting systematic reviews developed by Delphi consensus. J Clin Epidemiol.

[B19] Higgins JP, Thompson SG (2002). Quantifying heterogeneity in a meta-analysis. Stat Med.

[B20] Iglesias C, Torgerson D (2000). Does length of questionnaire matter? A randomised trial of response rates to a mailed questionnaire. J Health Serv Res Policy.

[B21] Dorman PJ, Slattery J, Farrell B, Dennis MS, Sandercock PA (1997). A randomised comparison of the EuroQol and Short Form-36 after stroke. United Kingdom collaborators in the International Stroke Trial. BMJ.

[B22] Dunn KM, Jordan K, Croft PR (2003). Does questionnaire structure influence response in postal surveys?. J Clin Epidemiol.

[B23] Jenkinson C, Coulter A, Reeves R, Bruster S, Richards N (2003). Properties of the Picker Patient Experience questionnaire in a randomised controlled trial of long versus short form survey instruments. J Public Health Med.

[B24] Jones R, Zhou M, Yates WR (2000). Improving return rates for health-care outcome. Psychol Rep.

[B25] Ward J, Boyle C, Long D, Ovadia C (1996). Patient surveys in general practice: a randomised trial of an instant lottery ticket to increase return rate. Aust Fam Physician.

[B26] Leigh Brown AP, Lawrie HE, Kennedy AD, Webb JA, Torgerson DJ, Grant AM (1997). Cost effectiveness of a prize draw on response to a postal questionnaire: results of a randomised trial among orthopaedic outpatients in Edinburgh. J Epidemiol Community Health.

[B27] Evans BR, Peterson BL, Demark-Wahnefried W (2004). No Difference in Response Rate to a Mailed Survey among Prostate Cancer Survivors Using Conditional versus Unconditional Incentives. Cancer Epidemiol Biomarkers Prev.

[B28] McColl E, Eccles MP, Rousseau NS, Steen IN, Parkin DW, Grimshaw JM (2003). From the generic to the condition-specific?: Instrument order effects in Quality of Life Assessment. Med Care.

[B29] Tai SS, Nazareth I, Haines A, Jowett C (1997). A randomized trial of the impact of telephone and recorded delivery reminders on the response rate to research questionnaires. J Public Health Med.

[B30] Salim Silva M, Smith WT, Bammer G (2002). Telephone reminders are a cost effective way to improve responses in postal health surveys. J Epidemiol Community Health.

[B31] Sutherland HJ, Beaton M, Mazer R, Kriukov V, Boyd NF (1996). A randomized trial of the total design method for the postal follow-up of women in a cancer prevention trial. Eur J Cancer Prev.

[B32] Parkes R, Kreiger N, James B, Johnson KC (2000). Effects on subject response of information brochures and small cash incentives in a mail-based case-control study. Ann Epidemiol.

[B33] Sackett DL, Straus SE, Richardson WS, Rosenberg W, Haynes RB (2000). Evidence-based medicine: how to practice and teach EBM.

[B34] Meinert CL, Tonascia S (1986). Clinical Trials: Design, Conduct and Analysis.

[B35] Ludemann R, Watson DI, Jamieson GG (2003). Influence of follow-up methodology and completeness on apparent clinical outcome of fundoplication. Am J Surg.

[B36] Heberlein TA, Baumgartner R (1978). Factors Affecting Response Rates to Mailed Questionnaires: A Quantitative Analysis of the Published Literature. American Sociological Review.

[B37] Kerlinger RN (1975). Foundations of behavioral research.

[B38] Edwards P, Roberts I, Sandercock P, Frost C (2004). Follow-up by mail in clinical trials: does questionnaire length matter?. Control Clin Trials.

[B39] Bowling A (1997). Research Methods in Health.

[B40] Harrison RA, Holt D, Elton PJ (2002). Do postage-stamps increase response rates to postal surveys? A randomized controlled trial. Int J Epidemiol.

[B41] Kalantar JS, Talley NJ (1999). The effects of lottery incentive and length of questionnaire on health survey response rates: a randomized study. J Clin Epidemiol.

